# Falls and Fall-Related Injuries among Community-Dwelling Adults in the United States

**DOI:** 10.1371/journal.pone.0150939

**Published:** 2016-03-15

**Authors:** Santosh K. Verma, Joanna L. Willetts, Helen L. Corns, Helen R. Marucci-Wellman, David A. Lombardi, Theodore K. Courtney

**Affiliations:** 1 Center for Injury Epidemiology, Liberty Mutual Research Institute for Safety, Hopkinton, MA, United States of America; 2 Department of Family Medicine and Community Health, University of Massachusetts Medical School, Worcester, MA, United States of America; 3 Environmental and Occupational Medicine and Epidemiology Program, Department of Environmental Health, Harvard T.H. Chan School of Public Health, Boston, MA, United States of America; Purdue University, UNITED STATES

## Abstract

**Introduction:**

Falls are the leading cause of unintentional injuries in the U.S.; however, national estimates for all community-dwelling adults are lacking. This study estimated the national incidence of falls and fall-related injuries among community-dwelling U.S. adults by age and gender and the trends in fall-related injuries across the adult life span.

**Methods:**

Nationally representative data from the National Health Interview Survey (NHIS) 2008 Balance and Dizziness supplement was used to develop national estimates of *falls*, and pooled data from the NHIS was used to calculate estimates of *fall-related injuries* in the U.S. and related trends from 2004–2013. Costs of unintentional fall-related injuries were extracted from the CDC’s Web-based Injury Statistics Query and Reporting System.

**Results:**

Twelve percent of community-dwelling U.S. adults reported falling in the previous year for a total estimate of 80 million *falls* at a rate of 37.2 falls per 100 person-years. On average, 9.9 million *fall-related injuries* occurred each year with a rate of 4.38 fall-related injuries per 100 person-years. In the previous three months, 2.0% of older adults (65+), 1.1% of middle-aged adults (45–64) and 0.7% of young adults (18–44) reported a fall-related injury. Of all fall-related injuries among community-dwelling adults, 32.3% occurred among older adults, 35.3% among middle-aged adults and 32.3% among younger adults. The age-adjusted rate of fall-related injuries increased 4% per year among older women (95% CI 1%–7%) from 2004 to 2013. Among U.S. adults, the total lifetime cost of annual unintentional fall-related injuries that resulted in a fatality, hospitalization or treatment in an emergency department was 111 billion U.S. dollars in 2010.

**Conclusions:**

Falls and fall-related injuries represent a significant health and safety problem for adults of all ages. The findings suggest that adult fall prevention efforts should consider the entire adult lifespan to ensure a greater public health benefit.

## Introduction

Advances in medicine, healthier and safer lifestyles, and technological developments have helped reduce morbidity and mortality in the U.S. and worldwide, with substantial declines in deaths due to communicable and non-communicable diseases such as cardiovascular disease and cancer, and motor vehicle traffic-related injuries.[1, 2] However, injuries due to falls, the leading cause of non-fatal injuries and the third leading cause of fatal injuries in the U.S., have been increasing in recent years.[2–7] In 2010, 37% of all medically consulted injury and poisoning episodes were due to falls.[6, 8] The Global Burden of Disease Study 2010 reported that between 1990 and 2010 Falls increased in rank from the 24^th^ to the 15^th^ leading cause of U.S. disability-adjusted life years (DALYs) with over a 50% increase in DALYs.[9, 10]

Primary prevention would be the most effective approach to reducing the burden of fall-related injuries. Several research studies and prevention programs have focused on fall prevention and examined *falls* as their main outcome of interest.[11] However, important baseline data, including a national estimate of the incidence of falls among community-dwelling U.S. adults, is lacking. Data from cohort studies on falls among older adults are cited widely.[12–14] However, data on fall incidence among young and middle-aged adults (working age adults) is rare.

In addition, despite being the leading cause of injury-related emergency department visits in nearly every adult age stratum, there remains a lack of detailed national estimates of *fall-related injuries* by age and gender. Using the National Health Interview Survey (NHIS), we estimated the national incidence of falls, the incidence of injuries resulting from falls, injury types, and the trend over time by age and gender among community-dwelling U.S. adults. As another measure of burden, we also report the national cost burden of fall-related injuries by age-group using the Centers for Disease Control (CDC) and Prevention’s Web-based Injury Statistics Query and Reporting System (WISQARS).

## Methods

The NHIS is an in-person household survey which is the principal source of information on the health of the civilian, noninstitutionalized population of the United States.[15] For each sampled household in the NHIS, interviews are conducted with an adult resident who answers questions related to the demographic, personal and health status characteristics of each member of the household.[16–18] The NHIS also surveys one Sample Adult (18+, SA) from each household on more detailed health and lifestyle topics. This study utilized existing publically available NHIS data and was exempted from Institutional Review Board approval.

### Fallers and Falls (NHIS 2008 Supplement)

In 2008, with sponsorship by the National Institute on Deafness and Other Communication Disorders (NIDCD), the SA Core of the NHIS included questions relating to balance/fall issues in adults in the previous 12 months. We defined Fallers (adults who experienced at least one fall in the previous 12 months) and Non-Fallers and the number of falls in the previous 12 months, regardless of injury, using the 2008 Balance and Dizziness supplement. Fallers and number of falls were determined by following questions:

1. “During the past 12 months, have you fallen at least once a month on average?”
2. “During the past 12 months, how many times have you fallen?”

SA who responded “yes” to the first question (*During the past 12 months*, *have you fallen at least once a month on average*?) were designated Fallers and their number of falls in the past 12 months was set to 12, and they were not asked the second question.

SA who responded “no” to the first question were then asked the second question (*During the past 12 months*, *how many times have you fallen*?). SA responding “0” to the second question were designated as Non-Fallers; and SA responding “1 time,” “2 times,” “3–4 times,” “5–7 times,” and “8 or more times” to the second question were designated as Fallers and the number of falls in the past 12 months were quantified as 1, 2, 3.5, 6, and 10, respectively, to calculate the total number of falls and the rate of falls per 100 persons.

In 2008, out of 20,752 sampled adults in the NHIS, 2,549 reported a fall in the previous year. The response rate in 2008 was 63%.

### Persons Injured from a Fall-Related Injury Episode and Total Fall-Related Injury Episodes (NHIS Annual Survey 2004–2013)

The NHIS collects information on medically consulted injury or poisoning episodes in the 3 months prior to the interview. Episodes are coded using the Ninth Revision of the International Classification of Disease external cause codes (ICD-9-CM). Persons experiencing a fall-related injury episode and the number of fall-related injury episodes between 2004 and 2013 were identified using ICD-9-CM codes: E880 -E888, “Accidental Falls” and ICD-9-CM codes 800–999, “Injury and Poisoning” were used to identify injury types. All analyses were performed using the Sample Adult subset to avoid proxy responses.

Over the study period (2004–2013), out of 289,187 sampled adults in the NHIS, 3,408 reported a fall-related injury in the previous three months. The average response rate was 66% during this period.

### Cost of Fall-Related Injuries

Costs of unintentional fatal and non-fatal fall-related injuries were extracted for the year 2010 from the CDC’s Web-based Injury Statistics Query and Reporting System (WISQARS).[19] The estimated cost is based on two primary components: 1) U.S. national frequency of injury fatality, hospitalization, and emergency department visits for the year 2010, and 2) unit (per case) lifetime medical and work loss cost estimates expressed in year 2010 U.S. prices. The methodology used to develop these unit cost estimates is documented in the Pacific Institute for Research and Evaluation methods report and by Finkelstein et al.[20] A detailed description of the calculations used to estimate total and average costs associated with injury-related deaths, hospitalizations, and emergency department visits can be found at http://www.cdc.gov/injury/wisqars/cost_help/calculations_toc.html. The total lifetime costs of unintentional fall-related injuries that resulted in a fatality, hospitalization or treatment in an emergency department in each age group were divided by the total population in the age group to calculate lifetime costs of fall-related injuries per person.

### Data Analysis

Using sample weights available in the NHIS data and the PROC SURVEYMEANS procedure in SAS 9.4, we estimated: 1) the number and proportion of adults (18+ years of age) who reported a *fall* in the past 12 months, 2) the number of *falls* experienced by adults in the past 12 months and the rate of *falls* per 100 person-years, 3), the number and proportion of adults 18+ reporting a *fall-related injury* in previous 3 months, and 4) the number of *fall-related injuries* to adults 18+ in the previous three months and the rate of *fall-related injuries* per 100 person-years. We annualized the estimates obtained from data pooled over multiple years (2004–2013). We examined the trend in the rate of *fall-related injuries* from 2004 to 2013 for 6 age-gender groups (18–44, 45–64, 65+; male and female). Poisson generalized linear models which accounted for sampling weight and clustering were used to examine the association between survey year and the rate of fall-related injuries in each age-gender group. The models were adjusted for age to account for change in average age by survey year in each age-gender group. We also estimated the number and proportion of injury types in these six age-gender groups.

## Results

### Fallers and Falls

Based upon the data from the NHIS 2008 supplement, 11.9% of community-dwelling U.S. adults fell in the previous 12 months (Table 1). The proportion of Fallers in the previous 12 months was 10.6% in the 18–44 year age-group, 11.4% in the 45–64 year age-group and 16.4% in the 65+ age-group. An estimated 80 million falls were reported in the prior year at a rate of 37.2 falls per 100 person-years. Among middle-aged (45–64 year age-group) and older adults (65+), 26.56 million (95% CI 23.01–30.12) and 17.05 million (95% CI 14.29–19.81) falls were reported, respectively.

**Table 1:** Number and Proportion of Community-dwelling Adults who Experienced One or More Falls in the Previous 12 Months and Number and Rate of Falls per 100 Person-Years by Age and Gender Groups.

		NHIS 2008 Fallers		NHIS 2008 Falls	
Gender	Age-Groups	Number of Persons (000s)^a^	Proportion per 100 Persons in the Previous 12 Months^b^ (95% CI)	Number of Falls (000s)^c^	Incidence Rate per 100 Persons in the Last 12 Months^d^ (95% CI)
**Overall**		**25,654**	**11.93(11.27–12.59)**	**80,007**	**37.21 (34.30–40.11)**
Females					
	**Overall**	**15,125**	**13.61(12.75–14.48)**	**45,316**	**40.79 (36.81–44.77)**
	18–24	1,794	12.89 (10.89–14.89)	6,712	48.25 (37.48–59.01)
	25–34	2,253	11.70 (10.02–13.38)	6,041	31.36 (24.55–38.17)
	35–44	2,343	11.57 (9.94–13.21)	7,683	37.95 (30.10–45.79)
	45–54	2,587	12.15 (10.33–13.96)	8,559	40.18 (31.37–49.00)
	55–64	2,533	15.49 (13.31–17.66)	7,008	42.84 (34.87–50.81)
	65–74	1,553	15.20 (12.75–17.66)	4,354	42.62 (30.89–54.36)
	75+	2,062	21.02 (18.44–23.60)	4,960	50.56 (40.81–60.31)
Males					
	**Overall**	**10,529**	**10.13 (9.28–10.98)**	**34,691**	**33.38 (29.76–37.00)**
	18–24	1,366	9.86 (7.88–11.85)	5,322	38.44 (28.86–48.02)
	25–34	1,715	8.91 (7.36–10.47)	5,140	26.71 (19.99–33.44)
	35–44	1,803	9.15 (7.43–10.88)	5,498	27.91 (20.38–35.44)
	45–54	1,811	8.85 (7.33–10.37)	5,831	28.51 (21.87–35.15)
	55–64	1,642	10.75 (8.94–12.56)	5,165	33.81 (25.30–42.32)
	65–74	1,066	12.11 (9.92–14.31)	3,637	41.32 (28.18–54.46)
	75+	1,127	17.05 (14.18–19.92)	4,097	61.97(49.48–74.46)

^a^Estimated frequency of people who reported falling in past 12 months (NHIS, 2008), in thousands.

^b^Estimated proportion (per 100 persons) of persons who fell in past 12 months (NHIS, 2008)

^c^Estimated frequency of falls in the past 12 months (NHIS, 2008), in thousands.

^d^Estimated incidence rate (per 100 persons) of falls in past 12 months (NHIS, 2008)

The proportion of male and female Fallers generally increased with each decade of age range after the 25–34 year age-group (Table 1). Similarly, the rate of falls increased with age after the 25–34 year age-group. Point estimates for fall rates were higher for women than men within each age stratum except in the 75+ age-group (95% CI overlapped for all the age-groups).

### Persons Injured from a Fall and Total Fall-Related Injury Episodes

Based upon this pooled NHIS annual survey data (2004 to 2013), 1.04% of community-dwelling U.S. adults reported a fall-related injury in the previous three months, resulting in approximately 9.9 million fall-related injuries annually (Table 2). An estimated 3.2 million fall-related injuries occurred among young adults (18–44 years), 3.5 million among middle-aged adults (45–64 years) and 3.2 million in older adults (65+).

**Table 2:** Number and Proportion of Community-dwelling Adults who Experienced a Fall-related Injury in the Previous 3 Months, Average Number of Fall-related Injuries in One Year and the Rate of Fall-related Injuries per 100 Person-Years in the U.S. NHIS 2004–2013.

		Persons Injured Due to a Fall		Injury Episodes from a Fall	
Gender	Age-Groups	Number (000s)^a^	Proportion per 100 Persons per Three Months (95% CI))^b^	Number (000s)^c^	Incidence Rate (per 100 Persons per Year (95% CI))^d^
**Overall**		**2,348**	**1.04 (0.99–1.08)**	**9,914**	**4.38 (4.18–4.58)**
Females					
	**Overall**	**1,487**	**1.27 (1.20–1.34)**	**6,278**	**5.36 (5.08–5.65)**
	18–24	111	0.76 (0.60–0.93)	451	3.11 (2.44–3.78)
	25–34	141	0.70 (0.59–0.81)	586	2.90 (2.44–3.35)
	35–44	168	0.80 (0.67–0.93)	730	3.49 (2.87–4.11)
	45–54	262	1.18 (1.05–1.31)	1,120	5.05 (4.50–5.61)
	55–64	278	1.57 (1.37–1.77)	1,183	6.69 (5.85–7.52)
	65–74	211	1.89 (1.66–2.12)	877	7.85 (6.86–8.85)
	75+	316	3.04 (2.72–3.37)	1,330	12.8 (11.43–14.17)
Males					
	**Overall**	**861**	**0.79 (0.73–0.84)**	**3,636**	**3.33 (3.10–3.57)**
	18–24	105	0.72 (0.54–0.90)	430	2.94 (2.21–3.66)
	25–34	108	0.54 (0.43–0.65)	447	2.22 (1.76–2.68)
	35–44	135	0.67 (0.55–0.78)	563	2.77 (2.29–3.26)
	45–54	150	0.71 (0.57–0.84)	663	3.13 (2.50–3.76)
	55–64	127	0.77 (0.64–0.90)	533	3.25 (2.69–3.80)
	65–74	95	0.99 (0.80–1.19)	412	4.29 (3.35–5.24)
	75+	140	2.04 (1.64–2.44)	588	8.55 (6.88–10.23)

^a^Estimated average frequency of persons reporting a medically-consulted injury due to a fall in past 3 months (NHIS, 2004–2013), in thousands.

^b^Estimated average incidence rate (per 100 persons) of persons who reported an injury episode due to a fall in past 3 months (NHIS, 2004–2013)

^c^Estimated average number of injury episodes due to a fall reported in past 3 months, annualized to one year (NHIS, 2004–2013), in thousands.

^d^Estimated average incidence rate (per 100 persons) of injury episodes resulting from a fall in past 3 months, annualized to one year (NHIS, 2004–2013)

The proportion of persons injured due to a fall increased monotonically with age after the 25–34 year age-group (Table 2). In the previous three months, 2.0% of older adults, 1.1% of middle-aged adults and 0.7% of young adults reported a fall-related injury. The rate of fall-related injuries also increased with age after the 25–35 year age group, and women over 75 years of age had the highest rate of fall-related injuries.

**Fig 1:**
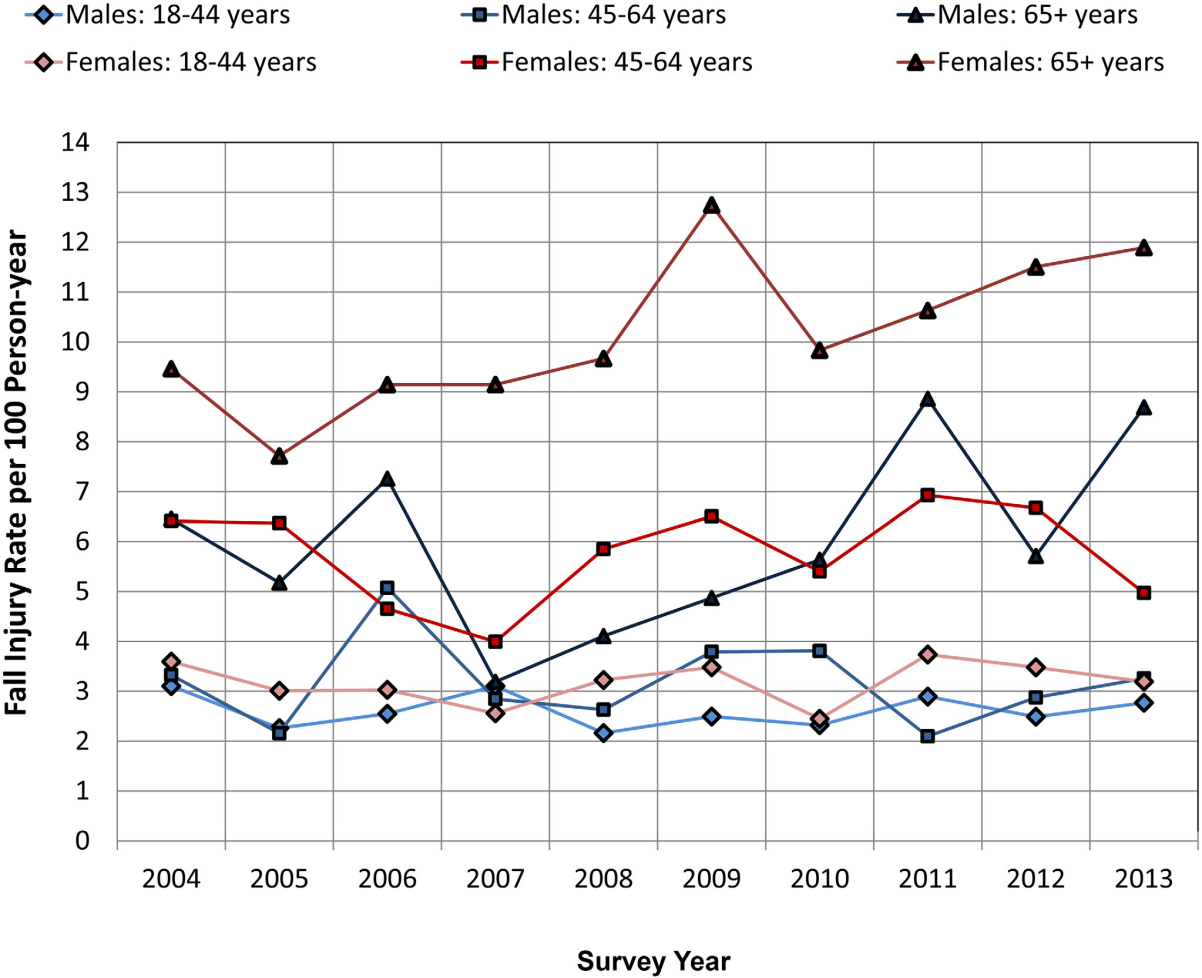
Incidence Rate of Fall-related Injuries per 100 Person-year by Age-Gender Groups from 2004 to 2013 in the U.S. Medically consulted fall-related injury episodes occurring in the previous three months among community-dwelling adults were identified in the National Health Interview Survey. We annualized the fall-related injury estimates and calculated rates of fall-related injuries per 100 Person-year by age and gender groups using population weights.

Fig 1 presents trends in the rates of fall-related injuries for community-dwelling adults from the pooled NHIS data by age-gender groups. Females and males in the 18–44 year age-groups had the lowest rate of fall-related injuries, and females over 65 years of age had the highest rate of fall-related injuries. The rates of fall-related injuries among females in the 45–64 year age-group and males over 65 years of age were very similar. Among all adults (18+), the increasing trend of the rate of fall-related injuries (after adjusting for age and gender) was 1% per year (95% CI 0%–3%, p < .10, not statistically significant at 0.05 level) over the survey years. Of the six age-gender groups, the age-adjusted rate of fall-related injuries increased 4% per year among older women (95% CI 1%–7%, p < .05) and 5% among older men (5% per year, 95% CI 0% -10%, p < .10, not statistically significant at 0.05 level) from 2004–2013.

In the overall adult population, the most common fall-related injury was “Sprains And Strains Of Joints And Adjacent Muscles” (38%, Table 3). However, among older adults, “Contusion With Intact Skin Surface” was the most common injury type (31% among males 65+, and 38% among females 65+). The proportion of fall-related injury “Open Wounds” was higher among men as compared to women. In the overall community-dwelling adult population, 28% of the fall-related injuries were fractures.

**Table 3:** Annual Average Fall-related injury^a^ types in thousands (000s) and their Proportion^b^ ^c^ by Age and Gender Groups. NHIS 2004–2013.

Nature of Injury (ICD-9 Code)	All Persons (18+) n,(%)	Males: 18–44 n,(%)	Males: 45–64 n,(%)	Males: 65+ n,(%)	Females: 18–44 n,(%)	Females: 45–64 n,(%)	Females: 65+ n,(%)
Fractures (800–829)	2791 (28.2)	349 (24.3)	363 (30.3)	261 (26.1)	381 (21.5)	630 (27.4)	807 (36.6)
Dislocation (830–839)	183 (1.8)	71 (5.0)	15 (1.2)	5 (0.5)	58 (3.3)	18 (0.8)	16 (0.7)
Sprains And Strains Of Joints And Adjacent Muscles (840–848)	3722 (37.6)	663 (46.1)	435 (36.3)	278 (27.8)	923 (52.2)	962 (41.8)	462 (20.9)
Intracranial Injury, Excluding Those With Skull Fracture (850–854)	136 (1.4)	17 (1.2)	10 (0.9)	15 (1.6)	32 (1.8)	36 (1.6)	26 (1.2)
Open Wounds (870–897)	1212 (12.2)	207 (14.4)	200 (16.7)	208 (20.8)	126 (7.1)	233 (10.1)	238 (10.8)
Superficial Injury (910–919)	646 (6.5)	75 (5.2)	64 (5.3)	112 (11.2)	94 (5.3)	133 (5.8)	167 (7.6)
Contusion With Intact Skin Surface (920–924)	2710 (27.3)	176 (12.2)	292 (24.4)	306 (30.6)	393 (22.2)	710 (30.8)	832 (37.7)
Certain Traumatic Complications And Unspecified Injuries (958–959)	409 (4.1)	38 (2.7)	57 (4.8)	55 (5.5)	34 (1.9)	108 (4.7)	116 (5.3)
Total ^b^ ^d^	11809 (119.1)	1596 (111.1)	1436 (119.9)	1240 (124.1)	2041 (115.3)	2830 (123.0)	2664 (120.8)

^a^ Estimated average number of injury episodes due to a fall reported in past 3 months, annualized to one year (NHIS, 2004–2013), in thousands.

^b^ Proportions are calculated as Number of Fall-injury events resulting in an injury type ÷ Total number of fall-related injury events. Mutliple injuries may result from a fall-injury event and thus proportions add upto more than 100%.

^c^ Injury types with less than 1% proportion of all fall injuries are excluded from the table.

^d^ Total excludes injury types with less than 1% proportion of all fall injuries

### Lifetime Unintentional Fall-related Injury Costs (CDC’s WISQARS, 2010)

Table 4 presents average and total lifetime costs of annual unintentional fall-related injuries resulting in death, hospitalization or an emergency department visit in the U.S. by age-groups. Average cost values under the headings for Death, Hospitalization and Emergency department visits are the average lifetime cost per fall-related injury that resulted in those outcomes, respectively. Total cost values under those same headings are the total lifetime cost of all fall-related injuries that resulted in the respective outcomes. For example, the total cost of fall-related injuries that resulted in death is equal to the average lifetime cost per fall-related injury resulting in death multiplied by the total number of fall-related injuries that resulted in death (e.g. in 18–24 age group, fall-related injuries resulted in 189 deaths, 1,887,705*189 = 356,776,245). “Total Cost of Fall-related Injuries” is the sum of the total costs of fall injuries that resulted in death, hospitalization and emergency department visits. “Cost of Fall-Related Injury per Person” represents the lifetime cost of fall-related injury per person (Total cost of fall-related injury in an age group ÷ total population in the age group).

**Table 4:** Average and total lifetime cost (US $) of Annual Unintentional Fall-Related Injuries Resulting in Death, Hospitalization or an Emergency Department Visit in the U.S. by age-groups, 2010*.

	Death		Hospitalization		Emergency Department Visits				
Age-groups	Average Cost ^c^ ($)	Total Cost^a^ ^d^ ($,000)	Average Cost ^c^ ($)	Total Cost^a^ ^d^ ($,000)	Average Cost ^c^ ($)	Total Cost^a^ ^d^ ($,000)	Total Cost of Fall-related Injuries ^a^ ^e^ ($,000)	Population in Each Age-group (2010) ^b^	Cost of Fall-Related Injury per Person ^f^ (Total Cost/Person, $)
**18–24**	1,887,705	356,776	181,949	2,938,802	6,575	4,011,673	**7,307,251**	30,672,088	**238.24**
**25–34**	1,908,136	570,533	183,753	5,159,815	6,648	5,296,202	**11,026,550**	41,063,948	**268.52**
**35–44**	1,550,187	764,242	197,013	7,781,336	6,938	5,179,236	**13,724,814**	41,070,606	**334.18**
**45–54**	1,083,457	1,390,075	185,623	14,988,049	7,107	6,349,632	**22,727,756**	45,006,716	**504.99**
**55–64**	600,934	1,208,478	117,596	12,362,819	5,783	4,321,450	**17,892,747**	36,482,729	**490.44**
**65–74**	275,964	824,580	84,523	12,305,483	4,895	2,740,821	**15,870,884**	21,713,429	**730.92**
**75+**	109,387	2,041,268	61,866	17,218,034	4,107	2,746,470	**22,005,772**	18,554,555	**1186.00**
**All Adults (18+)**	276,036	7,155,952	104,909	72,754,338	6,102	30,645,484	**110,555,774**	234,564,071	**471.32**

* Data extracted from WISQARS http://www.cdc.gov/injury/wisqars/

^a^ In thousands

^b^ Census data from 2010

^c^ Average lifetime cost of fall-related injury that resulted in death, hospitalization or emergency department visit, respectively

^d^ Total lifetime cost of fall-related injury that resulted in death, hospitalization or emergency department visit, respectively (average cost * number of injuries)

^e^ Total cost of fall-related injuries (sum of the total lifetime cost of fall-related injury that resulted in death, hospitalization or emergency department visit

^f^ Lifetime cost of fall-related injury per person (Total cost of fall-related injuries in an age group ÷ total population in the age group)

Among U.S. adults, the total lifetime cost of annual unintentional fall-related injuries that resulted in a fatality, hospitalization or treatment in emergency department was 111 billion U.S. dollars (Table 4). The highest total lifetime cost of unintentional fall-related injuries was in the 45–54 year age-group (23 billion U.S. dollars, Table 4). The costs of fall-related injuries per person were $1186, $731, $490 and $505 for the 75+, 65–74, 55–64 and 45–54 years age-groups, respectively.

## Discussion

Using a special supplement of the NHIS, one of the largest national health surveys in the U.S., we found that 12% of community-dwelling U.S. adults reported falling in the previous year for a total estimate of 80 million *falls* at a rate of 37.2 falls per 100 person-years. While this is the best estimate available at the national level and demonstrates a significant exposure to falling, it is likely an underestimate of the number of individuals every year exposed to falls. Fall incidents, especially those without an injury, are difficult to recall over a 12-month period. The proportion of Fallers in the oldest age group (65+) was 16.4%. This estimate, for the aforementioned reasons, is comparably lower than other estimates for this age group; a number of prior studies have reported that about a third of community-dwelling adults over 65 years of age report falling in one year.[12–14]

We found that the proportion of Fallers in the previous 12 months was 10.6% in the 18–44 year age-group, and 11.4% in the 45–64 year age-group. For comparison, a study using the data from the Baltimore Longitudinal Study on Aging found that 18.5% of young adults (20–45 years), 21% of middle-aged adults (46–65 years) and 35% of older adults (65+) reported one or more falls in the previous two years.[21] In this case, the recall period was twice as long as in the current study, and these proportions are correspondingly about double the values we report. Mertz et al., using the data from the Aerobics Center Longitudinal Study, which included participants referred (by self or employer) for the purpose of fitness evaluation and lifestyle counselling, found that 20% of the participants aged 20–87 years reported falling during the past year.[22] Some of the estimates from longitudinal studies are higher than those we observed using the NHIS 2008 Balance and Dizziness Supplement sample. However, these studies also assessed smaller samples, were conducted at a local level, and did not provide a national estimate.

Regardless of the variability in the findings across studies and the values we estimated from the NHIS 2008 Balance and Dizziness Supplement, the number of community-dwelling adults who report falling is high and represents a significant public health concern for not only older adults but for adults of all ages. As the number and the rate of fall-related injuries have increased in recent years, it is possible that the number of falls and falls rates have also increased from 2008. Therefore, in addition to collecting surveillance information about fall-related injuries, it is essential to also regularly collect information on falls as the high frequency of falls makes it possible for national and state-based surveillance systems to provide stable estimates, even for smaller geographic regions and, thus, assess risk factors for falls and trends over time. In addition, an emphasis on falls also provides the opportunity to efficiently conduct prospective studies and randomized controlled trials to evaluate fall prevention strategies in older adults as well as among middle-aged and younger adults. Finally, regular assessment of falls across all ages may help in early interventions to reduce the risk of falls and, ultimately, prevent fall-related injuries.

About 9.9 million *fall-related injuries* were reported each year among community-dwelling U.S. adults at a rate of 4.38 fall-related injuries per 100 person-years, according to the pooled NHIS data from 2004 to 2013. Corso et al. also reported a similar rate of fall-related injuries in the U.S. (4.2 per 100 person-years) using data from the Medical Expenditure Panel Survey, Healthcare Cost and Utilization Project—Nationwide Inpatient Sample and National Electronic Injury Surveillance System—All Injury Program.[23] The Centers for Disease Control and Prevention’s (CDC) Vital and Health Statistics publication reported 9.8 million fall-related injuries among U.S. adults in the year 2012.[24]

In the previous three months, 2.0% of older adults, 1.1% of middle-aged adult and 0.7% of young adults reported a fall-related injury and the rate of fall-related injuries increased with age. In general, older adults are more vulnerable to falls and injuries resulting from them. The injuries from falls are also more severe in older adults. Therefore, the focus of research and fall prevention has been predominantly on the older adult population[11] with only a few studies examining falls in middle-aged and young adults in the general population.[21, 25–33] The number of middle-aged adults in the U.S. is presently higher than the number of older adults, and even with lower rates of fall-related injury, the number of fall-related injuries among middle-aged adults (45–64) was similar to, if not higher than, older adults (65+).

Middle-aged adults may progressively start to experience higher incidence of disease and medication use along with decreasing postural stability, proprioception, and balance.[34–37] Furthermore, in most developed countries, wage earnings peak when individuals are in their 40s and 50s and, of all age-groups, middle-aged adults have the highest average household income in the U.S.[38] Injuries and concomitant loss of income during this phase of the life course may lead to a significant economic impact at the individual and family level.

The cost of injury data from WISQARS showed that the cost of fall-related injuries per person among middle-aged adults was about half of the cost among older adults.[19] A recent study from CDC reported that Otago Exercise Program, Tai Chi, and Stepping On were cost-beneficial for older adults with a positive return on investment.[39] Given the lower average cost of fall-related injuries, these programs may not be cost-beneficial for middle-aged adults. Thus, there is a need for more efficient fall risk assessment approaches that take into account multiple risk factors for falls in addition to age to identify middle-aged adults who are at a higher risk of falls and fall-related injuries, and focus fall prevention interventions towards these high risk individuals. Broad-based low cost fall prevention interventions could also be implemented earlier in the adult lifespan. Improved muscle strength, balance, and proprioception developed and maintained during middle-age may be carried over when the individual gets older, aiding in the reduction of falls and fall-related injuries in the older adult population.[40]

We observed an increasing trend (age- and gender-adjusted) in the rate of fall-related injuries from 2004 to 2013 among community-dwelling U.S adults (p <0.10, not statisically significant at 0.05 level) and, particularly, among older adults. We observed a similar magnitude of increasing trend among older adults of both genders (4% per year for older women and 5% per year for older men). However, the trend was not statistically significant for older men at 0.05 level. There was a smaller estimated number of men in comparison to women among those in the 65+ year age group (22 million vs. 36 million, table 1), which could have led to a higher year-to-year variability in fall-related injury incidence rate among older men. Hu and Baker reported a 6% increase from 2001 to 2007 in the rate of emergency department visits due to falls among older adults.[41] Two recent reports also observed age adjusted increases in fall incidence and emergency department-treated and hospitalization fall-related injuries among older U.S. adults.[42, 43] It is unclear whether increased awareness has led to improved reporting or if a true increase in fall-related injuries is occurring. As falls and fall-related injuries are increasing beyond demographic changes, more research is needed to understand possible reasons. Addressing the burden of fall-related injuries will require improved identification of adults who are at a higher risk for falls with evidence-based fall risk assessment approaches and the development and implementation of effective fall prevention programs across the adult life span.

More research is also needed regarding built environment design characteristics which can lower the risk of falls and fall-related injuries. For example, recently published studies have highlighted the need for more research in the areas of changes in floor level, stairs, slip-resistance of floor surfaces, lighting and visual cues.[44, 45] Studies have also demonstrated that impact-absorbing flooring significantly reduces the risk of injury in the event of a fall.[46] Other strategies to attenuate the impact force, such as hip protectors and martial arts training, may also reduce the risk of hip fracture following a fall.[47–49] In addition, a few laboratory studies have reported that repeated-slip training focusing on dynamic stability and weight support may be an effective intervention in reducing falls after a slipping event.[50–52] Lastly, measures to increase bone strength may lead to a reduced risk of fracture given a fall event.[53–55]

### Strengths and Limitations with Regards to Falls Surveillance

An important strength of this study is that it uses data from a large-scale, nationally representative, population-based survey which is conducted through in-person interviews—the NHIS. The 2008 Balance and Dizziness Supplement provided a unique opportunity to estimate the incidence of falls among U.S adults at the national level. The 2008 supplement questions relating to falls in adults had a 12-month recall period. A one-year recall period could lead to lower reported falls, particularly falls that do not result in an injury, potentially underestimating the number of Fallers and falls reported in this study. Older adults may have more difficulty recalling a fall event. Cumming et al. found that older adults often do not recall falls that occurred during specific periods of time over the preceding 3 to 12 months.[56] Warner et al., using data from the NHIS, previously suggested a recall period of 5 weeks to adequately recall less severe injuries.[57] The Prevention of Falls Network Europe (ProFaNE) recommends prospective daily recording with a minimum of monthly reporting.[58] Daily recordings could be difficult to accommodate in current national surveillance systems. However, given the high incidence of falls, a recall period of one month could be evaluated for use in these systems.

In addition, in the 2008 supplement, the number of falls was stratified for those who fell 3 or more times. We used the mid-points of strata “3–4 times (3.5 falls),” “5–7 times (6 falls),” and “8 or more times (10 falls, a midpoint between 8 and 12 falls)” to assign the number of falls. For more than 12 falls in the previous year, the number of falls was set to 12. The assignment of the number of falls based on stratum may have led to overestimation or underestimation of the total number of falls. We conducted a sensitivity analysis using the lower and upper boundaries of each stratum to assign the number of falls and observed that changing the assignment protocol did not significantly affect the overall estimate of the number of falls.

The NHIS does not include institutionalized individuals. Elderly individuals who fall and get injured are more likely to be institutionalized [59, 60] and, thus, are more likely to be excluded from the study. In addition, the excluded institutionalized population is composed primarily of the population in correctional institutions and nursing homes (91% of the 4.1 million institutionalized people in U.S. Census 2000).[61] Older adults in nursing homes are at a higher risk of falls and fall-related injuries.[62] Therefore, estimates based on NHIS data may underestimate the burden of falls and fall-related injuries, and the underestimation may be particularly significant for older adults.

## Conclusion

This study provides national estimates of falls and fall-related injuries among community-dwelling U.S. adults across the adult life span. Based on the 2008 NHIS Balance and Dizziness Supplement and the pooled NHIS annual data, about 12% of community-dwelling U.S. adults fall every year, experiencing an approximate national total of 80 million falls, and 9.9 million fall-related injuries occur every year among community-dwelling U.S. adults at a rate of 4.38 injuries per 100 person-years. In the previous three months, 2.0% of older adults, 1.1% of middle-aged adult and 0.7% of young adults reported a fall-related injury. Of all fall-related injuries to adults, 32.3% occurred among older adults, 35.3% among middle-aged adults and 32.3% among younger adults. Age-gender adjusted rates of fall-related injuries are increasing in the U.S.; more research is needed to understand possible reasons and to develop and implement effective fall and fall-related injury prevention strategies across all ages to significantly reduce the burden of injuries from falls.
